# Which transfer day results in the highest live birth rate for PCOS patients undergoing in vitro fertilization?

**DOI:** 10.1186/s12884-023-06173-5

**Published:** 2023-12-16

**Authors:** Yuying Guo, Fangfang Dai, Bo Zheng, Linlin Tao, Tieqing Cui

**Affiliations:** 1 Xingtai Infertility Specialist Hospital/Xingtai Reproduction and Genetics Specialist Hospital, Xingtai City, Hebei Province China; 2 HEBEI INSTITUTE OF MECHANICAL AND ELECTRICAL TECHNOLOGY, Xingtai City, Hebei Province China

**Keywords:** PCOS, Live birth rate, D4, D5, Transfer days, Implantation rate

## Abstract

**Background:**

Polycystic ovary syndrome (PCOS) has unusual levels of hormones. The hormone receptors in the endometrium have a hostile effect and make the microenvironment unfavorable for embryo implantation. The use of gonadotropin stimulation during in vitro fertilization (IVF) may have an impact on embryo implantation and live birth rate. According to recent data, the clinical results of day 4 embryo transfer (D4 transfer) were on par with those of day 5 embryo transfer (D5 transfer) in IVF-ET. There are few studies comparing the outcomes of transplants with various etiologies and days. The purpose of this study was to determine which transfer day had the best result for PCOS patients undergoing IVF.

**Methods:**

This retrospective cohort study was conducted in the Xingtai Infertility Specialist Hospital between January 2017 and November 2021. A total of 1,664 fresh ART cycles met inclusion criteria, including 242 PCOS transfers and 1422 tubal factor infertility transfers.

**Conclusions:**

PCOS individuals had the highest live birth rate on D4 transferred. It was not need to culture embryos to blastocysts to optimize embryo transfer for PCOS women. This could be a novel approach to transplantation for PCOS.

## Background

Polycystic ovary syndrome (PCOS) is an endocrine disorder that affects many facets of fertility. It is the cause of 18–25% of couples’ infertility. More patients with PCOS than those without the condition need assisted reproduction techniques (ARTs) therapy [1]. Despite recent advances in ARTs, allowing the selection of high-quality embryos, the implantation rate and live birth rate remain low and have not increased enough during in Vitro Fertilization (IVF) in recent decades [2].

Several reports have shown that gonadotropin stimulation in IVF treatment may change the endometrium and embryo quality, which may affect the live birth rate and embryo implantation [3, 4]. Horcajadas et al. found that more than 200 genes showed a differential endometrial gene expression of more than 3-fold when controlled ovarian hyperstimulation (COH) and normal cycles were compared at hCG + 7 versus LH + 7 [5]. Enhanced MAGEA11 and AR-mediated transcriptional regulation might impact a correct endometrial decidualization response and delay endometrial decidualization in PCOS [6]. Qiao et al. [7] used High-density oligonucleotide microarrays with 21,571 genes and found that the majority of the genes showing altered expression were down-regulated in endometrial samples from PCOS patients, especially those genes associated with membrane functions, extracellular matrix components, adhesion, invasive growth, and the cytoskeleton.

According to recent data, elective single blastocyst and single morula embryo transfer (ET) during in vitro fertilization embryo transfer (IVF-ET) produced similar clinical results [8–10]. In addition to the cleavage and blastocyst ET, morula ET may serve as an alternative option for the clinician [11]. Even some laboratories believed that D4 ET can be chosen to avoid ET cancellation in D5 due to unfavorable conditions in the IVF laboratory, but the decremented quality of embryos for transfer and the decreased pregnancy rate must be taken into consideration [12]. Is there a difference between PCOS and no-PCOS in the number of days for the embryo transfer to be successful? If the best day for transfer can be determined, PCOS implantation and live birth rates would rise considerably. These should consider suitable embryo quality, embryo-endometrium synchronization, and optimization of gene expression changes during the implantation window for PCOS transplantation. This study aimed to fill this gap in the literature by evaluating the best transfer day for PCOS patients undergoing IVF.

## Methods

### Study design and subject screening

**Fig. 1:**
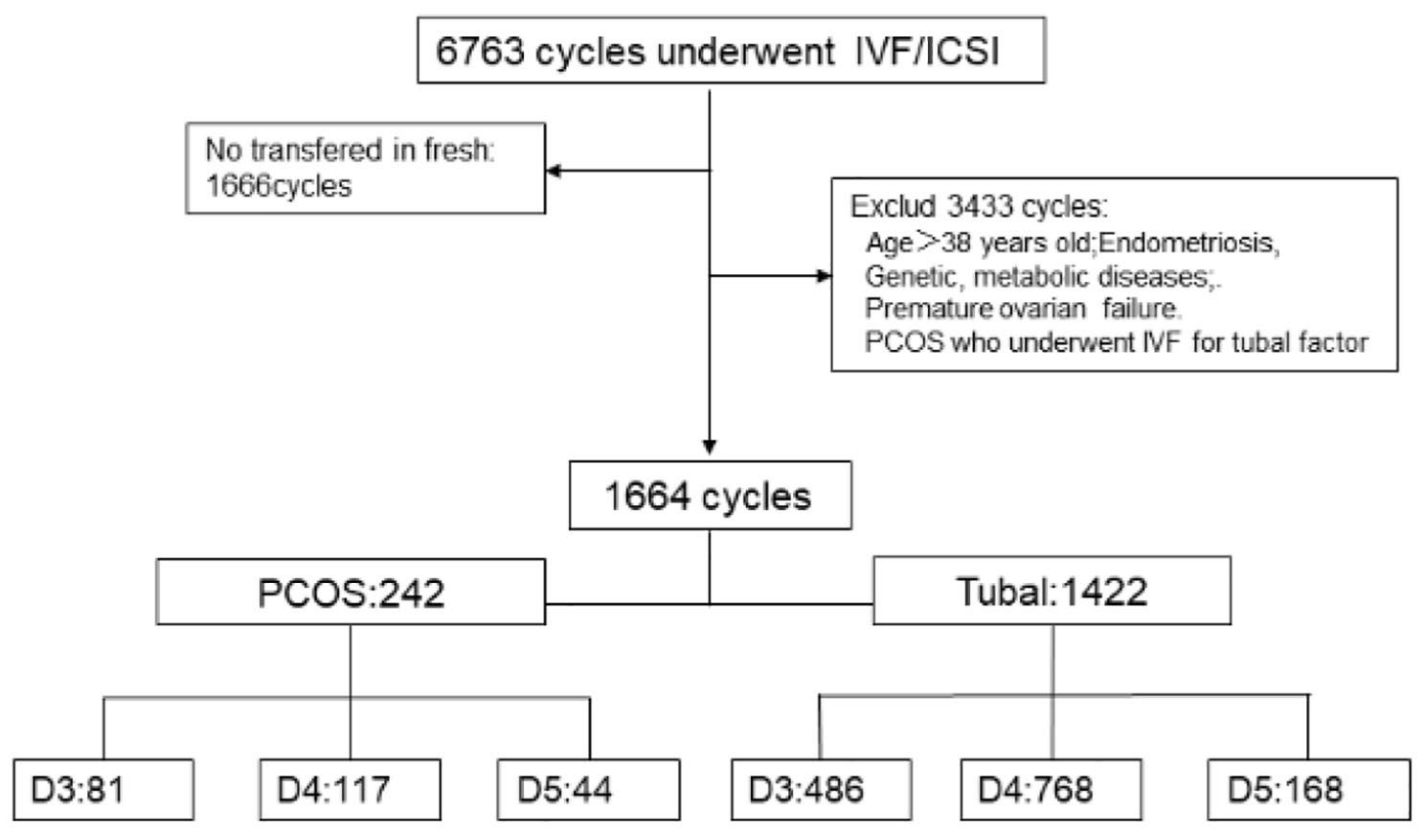
The flow chart of the study population

This retrospective cohort study was conducted in the Xingtai Infertility Specialist Hospital between January 2017 and November 2021. A total of 1,664 fresh ART cycles met inclusion criteria, including 242 PCOS transfers and 1422 tubal factor infertility transfers. We used the Rotterdam diagnostic Revised 2003 criteria (2 out of 3) in adults revised: (1) Oligo-or anovulation; (2) Clinical and/or biochemical signs of hyperandrogenism; (3) Polycystic ovaries and exclusion of other etiologies (congenital adrenal hyperplasia, androgen-secreting tumors, Cushing’s syndrome) [13]. The selection of the study patients is shown in Fig. 1.

The primary endpoint of this research was the fresh cycle clinical live birth rate, and the secondary endpoints were implantation rate, pregnancy rate, and miscarriage rate. There was no missing data in the study. Written approval for this study was obtained from the Ethics Board at Xingtai Reproduction and Genetics Specialist Hospital.

### Ovarian stimulation

All patients received GnRH agonist pituitary down-regulation protocol. 1.0 to 3.75 mg of long-acting Duffelin (IPsen, France) were administered. Subsequently, recombinant follicle stimulating hormone (r-FSH, Precon, Merck, Netherlands) was administered from 100 to 225 IU/d. Transvaginal ultrasonography was used to regularly measure follicle size, and blood levels of progesterone, LH, and E2 were used to measure follicle growth. When at least two leading follicles were ≥ 18 mm, 6000-10,000 IU of human chorionic gonadotropin (Zhuhai Lizon Pharmaceutical) was injected. 36–37 h later, the oocytes were taken out.

### Embryo culture

The oocyte collection and embryo culture system media used G-series (Vitrolife AB, Gothenburg, Sweden). All procedures were performed in standard incubators using 37℃, 6% CO_2_, and 5% O_2_ incubators.

After selective fertilization, one to two embryos were transplanted on D3/D4/D5 in accordance with the development of the embryos on D3 after fertilization. In our center, we grade embryos according to the 2011 ESHRE Istanbul Consensus and Ryh-sheng Li’s system [14], and blastocysts using the Gardner scoring system [15] as follows.

D3 embryos were assessed based on the number and blastomeres, the degree of fragmentation, and the presence of multinucleated cells. High-quality embryos were determined to have 7–8 cells, with 10% fragmentation. The available embryos were 6–10 cells, uneven blastomeres, and ≥ 10% fragmentation. They were both absence of multinucleated blastomeres.

D4 embryos, which lose all of the boundaries of the blastomere were classified as grade 1. Embryos with less than 100% but more than 50% of compaction will be rated as grade 2 [14]. The grade 1 was called high-quality embryo. And the grade 2 was called available-embryo.

The inner cell mass and trophectoderm quality of D5 embryos, whose growth and hatching status were assessed from grade 1 to grade 6, were both rated as A, B, and C [15]. The 4/5/6AA, 4/5/6AB, 4/5/6BA, and 4/5/6BB were called high-quality embryos. The 3AA/AB/BA/BB and 4/5/6BC/CB were called available embryos.

Women with overt ovarian hyperstimulation syndrome (OHSS) and thrombosis were not allowed to have embryo transplantation; The number of available embryos on the third day following fertilization determined which day to transfer. If the number of available embryos was equal to the number of transplants, the embryos were transplanted on D3. If the number of available embryos was higher than that of transplants, the early blastocysts or morulae were selected for transplantation on D4. The blastocysts were chosen and transplanted on D5 if there were more than four high-quality embryos available.

### Definition of clinical outcomes

Serum β-hCG was detected 12–14 days after transplantation to determine whether it was a biochemical pregnancy. About four weeks after transplantation, the beat of the gestational sac and the cardiac canal were observed by transvaginal ultrasound. The gestational sac was confirmed as a clinical pregnancy feature, and the number of gestational sacs was recorded.

### Statistical analysis

SPSS 27.0 (IBM) was used for statistical analyses. Continuous variables distribution was expressed as mean ± standard deviation and categorical variables were expressed as frequencies and percentages. Comparisons between groups were performed using one-way ANOVA, chi-square test, Fisher’s exact test, and nonparametric test. Logistic regression, presented as unadjusted odds ratio (crude odds ratio (OR)) or adjusted odds ratio (aOR) with a 95% confidence interval (CI), was performed. *P* < 0.05 was considered statistically significant.

## Results

### Patient characteristics

This retrospective cohort study was conducted in the Xingtai Infertility Specialist Hospital between January 2017 and November 2021. A total of 1,664 fresh ART cycles met inclusion criteria, including 242 PCOS and 1422 tubal factor transfers. Only patients who underwent fresh embryo transfer were included. The flowchart is shown in Fig. 1. The baseline characteristics of the PCOS and tubal groups are shown in Table 1. The age (*P* = 0.009) and AMH (*P* = 0.000) of the tubal group transferred on D3, D4, and D5 were different.

**Table 1:** Baseline characteristics of PCOS and tubal transferred cohorts on D3, D4, and D5

	PCOS			*P*-value	Tubal			*P*-value
	n = 242				n = 1422			
	D3	D4	D5		D3	D4	D5	
Cycles	81	117	44		486	768	168	
Age (years)	28.46 ± 3.97	29.00 ± 3.97	28.25 ± 3.50	0.421	30.26 ± 4.08*	30.83 ± 3.86**	30.01 ± 4.19	0.009
Type of infertility				0.100				0.317
Primary	69.14(56/81)	59.83(70/117)	72.73(32/44)		27.78(135/486)	26.95(207/768)	32.74(55/168)	
Secondary	30.86(25/81)	40.17(47/117)	27.27(12/44)		72.22(351/486)	73.05(561/768)	67.26(113/168)	
Infertility duration (year)	4.32 ± 2.93	4.63 ± 2.76	5.30 ± 2.98	0.192	3.48 ± 2.54	3.85 ± 2.90	3.52 ± 2.78	0.050
BMI (kg/m^2^)	25.06 ± 3.10	25.71 ± 3.16	25.96 ± 2.92	0.217	23.37 ± 3.08	23.77 ± 3.21	23.60 ± 2.94	0.089
FSH (mIU/mL)	6.01 ± 1.66	6.15 ± 1.79	5.93 ± 1.45	0.734	6.72 ± 2.25	6.71 ± 2.28	6.57 ± 1.70	0.730
AMH (ng/ mL)	7.55 ± 4.82	7.77 ± 3.92	7.91 ± 3.20	0.884	3.21 ± 2.12*	3.80 ± 2.28	3.93 ± 2.22***	0.000

* D3 *VS* D4 *P* <0.05; ** D4 *VS* D5 *P* <0.05; ***D3 *VS* D5 *P* <0.05;

BMI: body mass index; FSH: follicle-stimulating hormone; AMH: anti-Müllerian hormone;

### Clinical characteristics and outcomes

As can be seen from Table 2, the Gn amount (*P* = 0.001), number of oocytes (*P* = 0.003), E2 of HCG (*P* = 0.014), number of embryos per ET cycle (*P* = 0.000), and endometrium thickness (*P* = 0.000) were different among D3, D4, and D5 of PCOS. The E2 of HCG was lowest, while the endometrium thickness was thickest in the D4 PCOS group (all *P* < 0.05). The outcomes of PCOS were different in pregnancy rate (*P* = 0.001), implantation rate (*P* = 0.000), miscarriage rate (*P* = 0.011), and live birth rate (*P* = 0.000) among D3, D4, and D5; It is apparent from this table that the pregnancy rate and live birth rate of D4 were higher than D3 and D5 transferred patients with PCOS (all *P* < 0.05). From this data, we also could see that the number of oocytes (*P* = 0.000) and E2 of HCG (*P* = 0.000) of tubal on D5 was highest in the three group days; The implantation rates of tubal on D4 and D5 were both higher than D3 (all *P* < 0.05).

**Table 2:** Stimulation characteristics and outcomes of PCOS and tubal transferred cohorts on D3, D4, and D5

	PCOS			*P*-value	Tubal factor Infertility			*P*-value
	n = 242				n = 1422			
	D3	D4	D5		D3	D4	D5	
Cycles	81	117	44		486	768	168	
Gn(day)	11.84 ± 2.80	11.88 ± 2.91	11.41 ± 2.71	0.628	11.60 ± 1.61	11.59 ± 1.82	11.63 ± 1.46	0.954
Gn amount (IU)	2076.85 ± 767.48*	2530.41 ± 1016.26**	2114.77 ± 932.45	0.001	2511.86 ± 697.67	2537.42 ± 740.79	2451.04 ± 654.43	0.356
Number of oocytes	14.68 ± 5.06	13.87 ± 4.80**	16.98 ± 5.92***	0.003	11.44 ± 5.25*	12.64 ± 4.73**	14.63 ± 5.37***	0.000
Fertilization method				0.500				0.775
IVF	66.67(54/81)	65.81(77/117)	56.82(25/44)		81.48(396/486)	82.03(630/768)	83.93(141/168)	
ICSI	33.33(27/81)	34.19(40/117)	43.18(19/44)		18.52(90/486)	17.97(138/768)	16.07(27/168)	
Available embryo rate, %	43.86(325/741)	42.61(496/1164)	43.62(236/541)	0.846	45.00(1599/3553)	46.59(3222/6915)	46.02(873/1897)	0.303
E_2_ of HCG (pg/mL)	3276.37 ± 1569.93	3141.89 ± 1363.39**	3884.80 ± 1351.19***	0.014	2958.84 ± 1603.28*	3142.54 ± 1436.51**	3726.75 ± 1599.49***	0.000
P of HCG (ng/mL)	0.64 ± 0.31	0.59 ± 0.22	0.63 ± 0.25	0.351	0.81 ± 0.31	0.79 ± 0.27	0.83 ± 0.30	0.216
Endometrium thickness(mm)	10.49 ± 2.19*	11.82 ± 2.10**	11.37 ± 2.07***	0.000	11.29 ± 2.22	11.43 ± 2.14	11.7 ± 2.21	0.106
No. Of embryos per ET cycle	2.00 ± 0.00*	1.71 ± 0.46**	1.55 ± 0.50***	0.000	1.98 ± 0.16*	1.72 ± 0.45**	1.57 ± 0.50***	0.000
Pregnancy rate, %	51.85(42/81)*	76.92(90/117)**	61.36(27/44)	0.001	60.91(296/486)	63.02(484/768)	61.31(103/168)	0.735
Implantation rate, %	35.19(57/162)*	60(120/200)	55.88(38/68)***	0.000	41.88(402/960)*	49.01(646/1318)	53.41(141/264)***	0.000
Ectopic pregnancy rate, %	4.76(2/42)	1.11(1/90)	3.70(1/27)	0.418	1.69(5/296)	0.41(2/484)	1.94(2/103)	0.139
Miscarriage rate, %	35.71(15/42)*	13.33(12/90)	25.93(7/27)	0.011	15.54(46/296)	14.46(70/484)	16.50(17/103)	0.837
Multiple pregnancy rate, %	35.71(15/42)	33.33(30/90)	40.74(11/27)	0.777	35.47(105/296)	33.26(161/484)	36.89(38/103)	0.701
Live birth rate, %	32.10(26/81)*	65.81(77/117)**	43.18(19/44)	0.000	50.41(245/486)	53.65(412/768)	50.00(84/168)	0.452

* D3 *VS* D4 *P* <0.05; ** D4 *VS* D5 *P* <0.05; ***D3 *VS* D5 *P* <0.05;

A patient with D3 PCOS pregnancy had a live birth after intrauterine and extrauterine surgery

Gn: gonadotropin; IVF: in vitro fertilization; ICSI: intracytoplasmic sperm injection; P: progesterone

### Subgroup analysis

Due to the age of tubal and the oocyte number of the two groups being different among D3, D4, and D5, we stratified patients by age and different oocytes in Tables 3 and 4, and 5. Interestingly, there were also coincided differences in the live birth rate of D4 in ≤ 30 years women with PCOS. There was a higher live birth rate on D4 than on D3 and D5 of PCOS (all *P* < 0.05). Although there was no statistically significant difference in live birth rates of the > 30 years group patients with PCOS, a similar trend was presented; When the number of oocytes was less than or equal to 20, the live birth rate was different among D3, D4, and D5 (all *P* < 0.05). The implantation rates of tubal (*P* = 0.006 and *P* = 0.027) were different in the two age stages (≤ 30 years and >30 years groups). In the different oocytes from tubal groups, the implantation rates were different among D3, D4, and D5 in numbers between 11 and 15 (*P* = 0.014) and more than 20 oocytes (*P* = 0.009) groups. With between 11 and 15 tubal oocytes, D5 had a greater implantation rate than D3 and D4 (all *P* < 0.05); With more than 20 tubal oocytes, D4 and D5 had a higher implantation rate than D3 (all *P* < 0.05) (Table 5).

**Table 3:** Outcomes of ≤ 30 years women with PCOS and tubal transferred cohorts on D3, D4, and D5

	PCOS			*P*-value	Tubal factor Infertility			*P*-value
	n = 170				n = 705			
	D3	D4	D5		D3	D4	D5	
Cycles	58	80	32		257	351	97	
Age (years)	26.48 ± 2.50	27.11 ± 2.63	26.56 ± 2.21	0.295	27.06 ± 2.31	27.42 ± 2.40	27.06 ± 2.66	0.137
Infertility duration (year)	3.79 ± 1.86	4.15 ± 2.13	4.38 ± 2.46	0.885	3.17 ± 2.01	3.34 ± 2.14	3.39 ± 2.56	0.550
BMI (kg/m^2^)	25.05 ± 3.14	25.25 ± 3.10	25.56 ± 3.04	0.648	23.26 ± 3.25	23.55 ± 3.34	23.50 ± 3.21	0.549
Endometrium thickness(mm)	10.43 ± 2.08*	11.84 ± 2.01	11.05 ± 1.94	0.000	11.28 ± 2.16	11.48 ± 2.05	11.69 ± 2.26	0.216
Pregnancy rate, %	46.55(27/58)*	78.75(63/80)	62.50(20/32)	0.000	62.26(160/257)	66.10(232/351)	68.04(66/97)	0.489
Implantation rate, %	31.90(37/116)*	60.87(84/138)	56.25(27/48)***	0.000	44.34(227/512)*	51.72(315/609)	57.05(89/156)***	0.006
Ectopic pregnancy rate, %	7.41(2/27)	1.59(1/63)	5.00(1/20)	0.376	2.50(4/160)	0.86(2/232)	1.52(1/66)	0.430
Miscarriage rate, %	29.63(8/27)*	7.94(5/63)	20.00(4/20)	0.027	16.88(27/160)	14.66(34/232)	15.15(10/66)	0.834
Multiple pregnancy rate, %	37.04(10/27)	33.33(21/63)	35.00(7/20)	0.943	41.25(66/160)	35.34(82/232)	34.85(23/66)	0.446
Live birth rate, %	31.03(18/58)*	71.25(57/80)**	46.88(15/32)	0.000	50.19(129/257)	55.84(196/351)	56.70(55/97)	0.323

* D3 VS D4 *P* <0.05; ** D4 VS D5 *P* <0.05; ***D3 VS D5 P <0.05;

BMI: Body mass index

A patient with D3 PCOS pregnancy had a live birth after intrauterine and extrauterine surgery

**Table 4:** Outcomes of >30 years women with PCOS and tubal transferred cohorts on D3, D4, and D5

	PCOS			*P*-value	Tubal factor Infertility			*P*-value
	n = 72				n = 717			
	D3	D4	D5		D3	D4	D5	
Cycles	23	37	12		229	417	71	
Age (years)	33.86 ± 2.20	33.70 ± 2.12	34.04 ± 1.91	0.383	33.43 ± 2.25	33.08 ± 1.62	32.75 ± 1.91	0.578
Infertility duration (year)	3.82 ± 2.99	4.28 ± 3.35	3.70 ± 3.06	0.136	5.65 ± 4.43	5.68 ± 3.60	7.75 ± 2.93	0.228
BMI (kg/m^2^)	23.50 ± 2.87	23.96 ± 3.09	23.74 ± 2.56	0.169	25.10 ± 3.08	26.71 ± 3.10	27.03 ± 2.39	0.086
Endometrium thickness(mm)	10.59 ± 2.38	11.76 ± 2.23	12.23 ± 2.24	0.084	11.26 ± 2.29	11.35 ± 2.24	11.60 ± 2.26	0.550
Pregnancy rate, %	65.22(15/23)	72.97(27/37)	58.33(7/12)	0.601	59.39(136/229)	60.43(252/417)	52.11(37/71)	0.419
Implantation rate, %	43.48(20/46)	58.06(36/62)	55.00(11/20)	0.314	39.06(175/448)*	46.69(331/709)	48.15(52/108)	0.027
Ectopic pregnancy rate, %	0.00(0/15)	0.00(0/27)	0.00(0/7)	--	0.74(1/136)	0.00(0/252)	2.70(1/37)	0.070
Miscarriage rate, %	46.67(7/15)	25.93(7/27)	42.86(3/7)	0.355	13.97(19/136)	14.29(36/252)	18.92(7/37)	0.735
Multiple pregnancy rate, %	33.33(5/15)	33.33(9/27)	57.14(4/7)	0.481	28.68(39/136)	31.35(79/252)	40.54(15/37)	0.386
Live birth rate, %	34.78(8/23)	54.05(20/37)	33.33(4/12)	0.240	50.66(116/229)	51.80(216/417)	40.85(29/71)	0.232

* D3 VS D4 *P* <0.05;

**Table 5:** Outcomes of different oocytes retrieved from PCOS and tubal transferred on D3, D4, and D5

	PCOS			*P*-value	Tubal factor Infertility			*P*-value
	n = 242				n = 1422			
≤ 10 oocytes	D3	D4	D5		D3	D4	D5	
Cycles	17	33	5		231	287	39	
Pregnancy rate, %	35.29(6/17)	60.61(20/33)	60.00(3/5)	0.178	60.17(139/231)	63.76(183/287)	51.28(20/39)	0.285
Implantation rate, %	23.53(8/34)*	50.00(28/56)	57.14(4/7)	0.032	40.18(182/453)	47.64(242/508)	45.76(27/59)	0.065
Live birth rate, %	29.41(5/17)	54.55(18/33)**	0.00(0/5)	0.032	51.08(118/231)	53.31(153/287)	38.46(15/39)	0.219
11–15 oocytes								
Cycles	29	41	18		156	277	66	
Pregnancy rate, %	58.62(17/29)	75.61(31/41)	55.56(10/18)	0.196	62.18(97/156)	61.01(169/277)	68.18(45/66)	0.557
Implantation rate, %	43.10(25/58)	61.11(44/72)	53.57(15/28)	0.123	42.07(130/309)	47.33(230/486)**	58.18(64/110)***	0.014
Live birth rate, %	27.59(8/29)*	65.85(27/41)	38.89(7/18)	0.005	47.44(74/156)	52.35(145/277)	60.61(40/66)	0.195
16–20 oocytes								
Cycles	25	34	9		70	161	40	
Pregnancy rate, %	52.00(13/25)*	91.18(31/34)	77.78(7/9)	0.003	70.00(49/70)	69.57(112/161)	55.00(22/40)	0.186
Implantation rate, %	32.00(16/50)*	65.52(38/58)	75.00(12/16)***	0.000	52.86(74/140)	55.30(146/264)	49.15(29/59)	0.669
Live birth rate, %	36.00(9/25)*	73.53(25/34)	55.56(5/9)	0.016	60.00(42/70)	60.87(98/161)	40.00(16/40)	0.051
>20 oocytes								
Cycles	10	9	12		29	43	23	
Pregnancy rate, %	60.00(6/10)	88.89(8/9)	58.33(7/12)	0.272	37.93(11/29)	46.51(20/43)	69.57(16/23)	0.067
Implantation rate, %	40.00(8/20)	71.43(10/14)	41.18(7/17)	0.144	27.59(16/58)*	46.67(28/60)	58.33(21/36)***	0.009
Live birth rate, %	40.00(4/10)	77.78(7/9)	58.33(7/12)	0.249	37.93(11/29)	37.21(16/43)	56.52(13/23)	0.274

* D3 *VS* D4 *P* <0.05; ** D4 *VS* D5 *P* <0.05; ***D3 *VS* D5 *P* <0.05

**Fig. 2:**
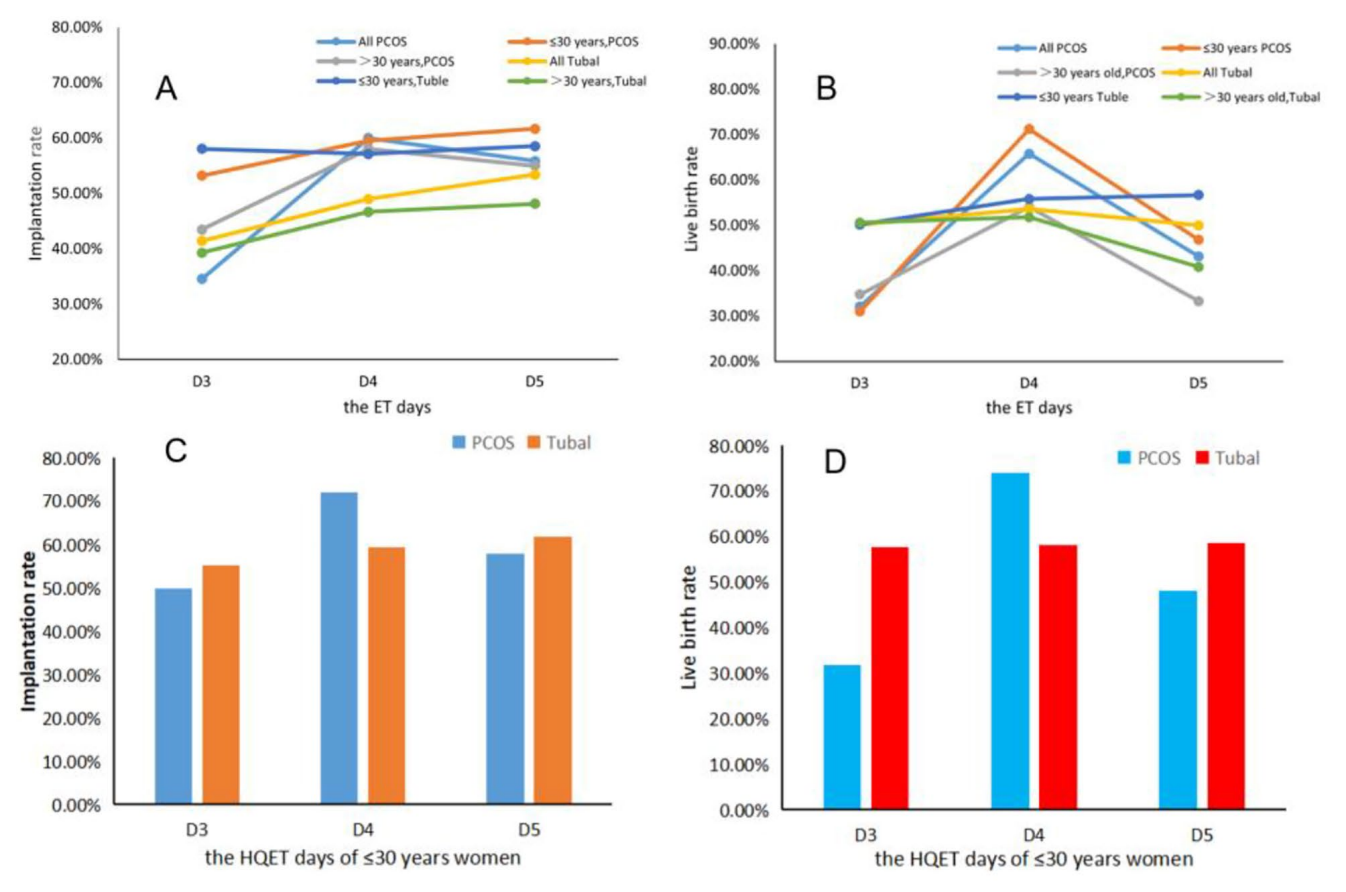
Live birth rate and implantation rate of PCOS and tubal transferred cohorts on D3, D4, and D5 HQET: high quality embryos transferred A: All PCOS, All Tubal, ≤ 30 years, PCOS, ≤ 30 years, Tuble and >30 years, Tubal, *P*<0.05 B: All PCOS and ≤ 30 years,PCOS, *P*<0.05 C: PCOS VS Tubal factor in D4, *P*<0.05 D: PCOS VS Tubal factor in D3 and D4, *P*<0.05

We further performed subgroup analysis by selecting high-quality embryos of ≤ 30 years women with PCOS and tubal in Table 6. Interestingly, the live birth rates were different in the three same-day transferred groups. The live birth rate of tubal was higher than PCOS on D3 (*P* = 0.032); The situation on D4 was the contrary, PCOS has a higher live birth rate than tuble (*P* = 0.031). There was no difference between them on D5 (Fig. 2).

**Table 6:** Outcomes of high quality embryos of ≤ 30 years women with PCOS and tubal transferred cohorts on D3, D4, and D5

	D3		*P*-value	D4		*P*-value	D5		*P*-value
	n = 100			n = 271			n = 97		
	PCOS	Tubal factor		PCOS	Tubal factor		PCOS	Tubal factor	
Cycles	22	78		54	217		27	70	
Age (years)	25.64 ± 2.52	26.13 ± 2.05	0.365	26.94 ± 2.82	27.24 ± 2.49	0.457	26.30 ± 2.16	27.21 ± 2.59	0.106
Infertility duration (year)	4.45 ± 2.18	2.55 ± 1.64	0.000	3.98 ± 1.95	3.31 ± 2.06	0.028	3.93 ± 2.38	3.23 ± 2.02	0.151
BMI (kg/m^2^)	24.95 ± 3.33	22.90 ± 3.35	0.015	25.41 ± 3.02	23.49 ± 3.25	0.000	25.58 ± 3.12	23.57 ± 3.21	0.007
Endometrium thickness(mm)	9.97 ± 2.05	10.93 ± 2.27	0.085	12.08 ± 2.00	11.58 ± 1.98	0.093	11.14 ± 2.07	11.85 ± 2.09	0.139
Pregnancy rate, %	54.55(12/22)	70.51(55/78)	0. 160	83.33(45/54)	70.51(153/217)	0.057	62.96(17/27)	70.00(49/70)	0.505
Implantation rate, %	40.91(18/44)	55.13(86/156)	0.095	72.09(62/86)	59.25(205/346)	0.028	57.89(22/38)	61.68(66/107)	0.681
Ectopic pregnancy rate, %	16.67(2/12)	1.82(1/55)	0.080	2.22(1/45)	0.65(1/153)	0.355	5.88(1/17)	2.04(1/49)	0.426
Miscarriage rate, %	33.33(4/12)	16.36(9/55)	0 0.228	8.89(4/45)	16.99(26/153)	0.183	17.65(3/17)	14.29(7/49)	0.739
Multiple pregnancy rate, %	50.00(6/12)	56.36(31/55)	0.688	37.78(17/45)	33.99(52/153)	0.639	29.41(5/17)	34.69(17/49)	0.691
Live birth rate, %	31.82(7/22)	57.69(45/78)	0. 032	74.07(40/54)	58.06(126/217)	0.031	48.15(13/27)	58.57(41/70)	0.354

A patient with D3 PCOS pregnancy had a live birth after intrauterine and extrauterine surgery

The associations of different transferred days and live birth rates of PCOS and tubal were shown in Table 7. What stands out in the table was the D4 of PCOS (unadjusted OR 2.533, 95% CI 1.247 to 5.143, *P* = 0.010; adjusted OR 3.328, 95% CI 1.532 to 7.229, *P* = 0.002) had a positively and significantly increased effort for live birth.

**Table 7:** Crude and adjusted odds ratios (ORs) of different transferred days for live birth rates

		OR	95%CI	*P*-value			OR	95%CI	*P*-value
PCOS	Crude				Tubal	Crude			
	D5	1.000				D5	1.000		
	D4	2.533	1.247–5.143	0.010		D4	1.157	0.829–1.616	0.319
	D3	0.622	0.292–1.326	0.219		D3	1.017	0.716–1.444	0.927
	Adjusted					Adjusted			
	D5	1.000				D5	1.000		
	D4	3.328	1.532–7.229	0.002		D4	1.194	0.851–1.675	0.304
	D3	0.679	0.300-1.535	0.352		D3	1.032	0.721–1.478	0.863

Adjusted: age, AMH, BMI, and endometrium thickness

## Discussion

According to this study, D4 transplanted patients had the highest live birth rate of PCOS patients. It might be not necessary to culture to blastocyst to optimize embryo transfer for PCOS women. This could be a novel way for PCOS patients to schedule their transplantation.

The primary goal of IVF-ET is the delivery of a healthy baby. ART has advanced over 40 years, yet the live birth rates remain poor [2]. PCOS patients are at a greater risk of pregnancy loss [16]. Many Pretreatment methods including GnRH agonist and hormone replacement treatment protocol, Metformin, and endometrial shedding with progestin before ovarian stimulation could not improve the live birth rate for PCOS women [17–19]. Low embryo quality, aberrant uterine receptivity, and abnormal trophoblast invasion in PCOS women can predispose to low implantation, miscarriage, high risks of adverse birth outcomes, and low live birth rate [20–22].

To select the most developing embryos, blastocyst culturing was used in IVF-ET. According to certain findings, transferring blastocyst-stage embryos increases the likelihood of a live delivery compared to transferring cleavage-stage embryos [23, 24]. However, there were significant drawbacks to blastocyst transfer, including a possible unbalanced sex ratio in favor of men and a higher risk of monozygotic twinning (MZT) [25]. According to recent data, elective single blastocyst and single morula embryo transfer in IVF-ET produced similar clinical results [8–10]. The morula-stage embryo may have similar advantages compared to the blastocyst-stage embryo because it has both the activated embryonic genome [26]. The importance of Day 4 embryos in IVF should not be undervalued according to the literature [27, 28]. D4 good morula embryo transfer resulted in a compatible live birth rate with D5 blastocyst embryo transfer in fresh IVF/ET cycles [14], as well as similar pregnancy rate in an integrated time-lapse incubator [29–31]. According to our research, transferred D4 and D5 of tubal or PCOS both had a higher implantation rate than D3 (all *P* < 0.05), which was consistent with before report [23]. We also found that the live birth rate of PCOS was higher on D4 than on D3 or D5 (all *P* < 0.05). However, there was no statistically significant difference in live birth rates among D3, D4, and D5 of tubal. The live birth rate of tubal was greater than PCOS on D3 and a lower live birth rate than PCOS on D4 of the same-day transferred groups in subgroup analysis by selecting high-quality embryos from women aged 30 years or younger (*P* < 0.05). The rate of live births differed between the tubal and PCOS. Recently, Wang et al. [32] found that the transfer of blastocyst-stage embryos was associated with shorter leukocyte telomere length (LTL) in children than the transfer of cleavage-stage embryos. It implied a better reason to transfer D4 than D5.

Successful implantation requires endometrial receptivity status for embryo localization, adhesion, invasion, and implantation. Notably, whether its tolerance synchronous alteration with the growth of the embryo is key to determining implantation. Synchronous coordination of the endometrial receptivity status and embryonic development is necessary for successful implantation.

In this study, we found the tubal and PCOS have differences in live birth rates undergoing IVF-ET. This may reflect the differences between the endometrial receptivity of tubal and PCOS during COH. It was reported that COH might negatively affect endometrial receptivity [33] for implantation [34]. Ovarian stimulation for IVF profoundly alters the luteal phase of endometrial development. Zapantis et al. [35] reported the premature appearance of the nucleolar channel system (NCS) and hence maturation of the endometrium following COH [36]. Exogenous gonadotropin in IVF decreased the implantation rate [37]. The mTOR signaling might be involved in the effect of maternal hyperinsulinemia on the endometrium receptivity process [38]. PCOS has unusual levels of hormones. The hormone receptors in the endometrium have a hostile effect and make the microenvironment unfavorable for embryo implantation. The endometrial receptivity of PCOS was closely associated with gene expression, energy metabolism, and endocrine environment [39]. The cell cycle was the fundamental biological process dysregulated in the endometrium of PCOS women, affecting decidualization progression in the endometrium during the implantation window [40]. Zhang et al. [41] discovered that as PCOS worsens, alterations in various signal pathways may decrease endometrial receptivity and reproductive outcomes.

Embryo-endometrium synchronization is important in embryo transfer. A study demonstrated abnormal gene expression in human PCOS oocytes. These genes included processes such as maternal effect genes and the mitotic cell cycle [42]. It has been shown that the time to initiate compaction and reach the morula stage as well as the duration of the 4th cleavage division was significantly shorter in PCOS embryos compared with non-PCOS embryos [43]. Moreover, it was observed that faster embryonic growth in PCOS preimplantation embryos, and there appear to be worse obstetric outcomes for these patients [44]. Given the maturity of endometrium following COH [36], it appeared that transfer on D4 was a good decision, particularly for PCOS.

A study thought that serum E2 levels on the day of trigger were not a good predictor of live birth rate or perinatal and obstetrical outcomes [45]. However, the other studies thought that peak E2 level on hCG trigger day was associated with the cumulative live birth rate (cLBR) in a segmental pattern [46]. Our results showed that the E2 of HCG among D3, D4, and D5 were all different in PCOS and tubal groups (all *P* < 0.05). Since estrogen matched with the number of oocytes retrieved, we chose the number of eggs collected for group analysis to avoid statistical errors. Our results showed that the implantation rate of tubal oocyte numbers between 11 and 15 were different in three transferred days (*P* = 0.014). D5 had a higher implantation rate than D3 and D4 of tubal oocytes between 11 and 15 (all *P* < 0.05); The live birth rate of PCOS was higher on D4 than D3 when oocytes number between 11 and 20 (all *P* < 0.05). The results of this study showed that D4 of PCOS (unadjusted OR 2.533, 95% CI 1.247 to 5.143, *P* = 0.010; adjusted OR 3.328, 95% CI 1.532 to 7.229, *P* = 0.002) had a positively and significantly increased effort for live birth. Those might imply that PCOS transferred on D4 were better, especially the live birth rate.

The outcomes of PCOS were different from tubal. The reasons for this conclusion may be: First, it should be noted that PCOS had a lower endometrial receptivity than tubal. Additionally, ovarian stimulation changed how the endometrial luteal phase developed and appeared to affect receptivity for implantation. The premature appearance of the NCS and hence maturation of the endometrium following COH [27]. Also, it has been reported that morulae and blastocysts have similar advantages. They both have activated embryonic genomes and are more synchronized with the endometrium than D3 cleavage embryos [39]. Added faster embryonic growth in PCOS preimplantation embryo [44], it could be the implant window of PCOS during COH earlier than the fallopian tube. The embryo quality, embryo-endometrial synchrony, and altered gene expression during the window of implantation were important for the implantation and live birth. Those might imply that PCOS transferred on D4 were better, especially the live birth rate. A prospective randomized control experiment is also required to verify the findings.

### Strengths and limitations

The main benefit of our study was that it investigated the outcomes of PCOS and oviduct factor infertility in different transferred days of D3, D4, and D5. This study does, however, have some limitations since it was a retrospective single-center study. Efforts to boost the patients’ pregnancy rate at the time, we transplanted more embryos than now when *P* ≤ 1.5 ng/mL (99.28% of the entire cycle). This resulted in a high multiple pregnancy rate. We have now been able to reduce the number of transplant as well as the multiple pregnancy rate since realizing this problem. Moreover, we cannot exclude bias due to different ethnicities. Further multi-center randomized clinical trials would be needed for confirmation. In addition, due to the nature of the study unknown confounding factors cannot be excluded. Prospective randomized control trials should be addressed in the future to investigate the best day for transfer.

## Conclusion

In conclusion, the current study showed that live birth rates of PCOS were different on various transferred days. The highest live birth rate of PCOS patients was on D4 transferred. These results suggested that it was not need to culture embryos to blastocysts to optimize embryo transfer for PCOS women. This may be a new transplantation strategy for PCOS.

## Data Availability

The datasets during the current study are available from the corresponding author on reasonable request.

## References

[1] Hart R, Doherty DA (2015). The potential implications of a PCOS diagnosis on a woman’s long-term health using data linkage. J Clin Endocrinol Metab.

[2] Lopes IM, Baracat MC, Simões Mde J, Simões RS, Baracat EC, Soares JM Jr. Endometrium in women with polycystic ovary syndrome during the window of implantation. Rev Assoc Med Bras (1992). 2011, 57(6):702-9. 10.1590/s0104-42302011000600020 22249553

[3] Mitter VR, Gradel F, Kohl Schwartz AS, von Wolff M. Gonadotropin Stimulation Reduces the Implantation and Live Birth Rate but Not the Miscarriage Rate of Embryos Transferred When Compared to Unstimulated In Vitro Fertilization. Reproductive sciences (Thousand Oaks, Calif). 2023, 30(1):283 – 90. 10.1007/s43032-022-01016-8 PMC9810560 35768691

[4] Orvieto R, Meltzer S, Rabinson J, Zohav E, Anteby EY, Nahum R (2008). GnRH agonist versus GnRH antagonist in ovarian stimulation: the role of endometrial receptivity. Fertil Steril.

[5] Horcajadas JA, Riesewijk A, Polman J, van Os R, Pellicer A, Mosselman S (2005). Effect of controlled ovarian hyperstimulation in IVF on endometrial gene expression profiles. Mol Hum Reprod.

[6] Younas K, Quintela M, Thomas S, Garcia-Parra J, Blake L, Whiteland H (2019). Delayed endometrial decidualisation in polycystic ovary syndrome; the role of AR-MAGEA11. J Mol Med (Berl).

[7] Qiao J, Wang L, Li R, Zhang X (2008). Microarray evaluation of endometrial receptivity in Chinese women with polycystic ovary syndrome. Reprod Biomed Online.

[8] Kang SM, Lee SW, Jeong HJ, Yoon SH, Koh MW, Lim JH (2012). Clinical outcomes of elective single morula embryo transfer versus elective single blastocyst embryo transfer in IVF-ET. J Assist Reprod Genet.

[9] Feil D, Henshaw Rc, Fau-Lane M, Lane M (2008). Day 4 embryo selection is equal to Day 5 using a new embryo scoring system validated in single embryo transfers. Hum Reprod.

[10] Skorupski JC, Stein DE, Acholonu U, Field H, Keltz M (2007). Successful pregnancy rates achieved with day 4 embryo transfers. Fertil Steril.

[11] Zhang HN, Ying YF, Xi HT, Lu XS, Zhao JZ, Chen YL (2021). Comparison of pregnancy outcomes between single-Morula embryo transfer and single-blastocyst transfer in fresh IVF/ICSI cycles. Med Sci Monit.

[12] Lee SH, Lee HS, Lim CK, Park YS, Yang KM, Park DW (2013). Comparison of the clinical outcomes of day 4 and 5 embryo transfer cycles. Clin Exp Reprod Med.

[13] Rotterdam ESHRE, ASRM-Sponsored PCOS consensus workshop group (2004). Revised 2003 consensus on diagnostic criteria and long-term health risks related to polycystic ovary syndrome (PCOS). Hum Reprod.

[14] Li RS, Hwu YM, Lee RK, Li SH, Lin MH (2018). Day 4 good morula embryo transfer provided compatible live birth rate with day 5 blastocyst embryo in fresh IVF/ET cycles. Taiwan J Obstet Gynecol.

[15] Gardner DK, Lane M (1997). Culture and selection of viable blastocysts: a feasible proposition for human IVF?. Hum Reprod Update.

[16] Yang W, Yang R, Lin M, Yang Y, Song X, Zhang J (2018). Body mass index and basal androstenedione are Independent risk factors for miscarriage in polycystic ovary syndrome. Reproductive Biology and Endocrinology: Reprod Biol Endocrinol.

[17] Liu X, Shi J, Bai H, Wen W (2021). Pretreatment with a GnRH agonist and hormone replacement treatment protocol could not improve live birth rate for PCOS women undergoing frozen-thawed embryo transfer cycles. BMC Pregnancy Childbirth.

[18] Tso LO, Costello MF, Albuquerque LET, Andriolo RB, Macedo CR (2020). Metformin treatment before and during IVF or ICSI in women with polycystic ovary syndrome. Cochrane Database Syst Rev.

[19] Diamond MP, Kruger M, Santoro N, Zhang H, Casson P, Schlaff W (2012). Endometrial shedding effect on conception and live birth in women with polycystic ovary syndrome. Obstet Gynecol.

[20] Palomba S, Piltonen TT, Giudice LC (2021). Endometrial function in women with polycystic ovary syndrome: a comprehensive review. Hum Reprod Update.

[21] Yu H, Liang Z, Cai R, Jin S, Xia T, Wang C (2022). Association of adverse birth outcomes with in vitro fertilization after controlling infertility factors based on a singleton live birth cohort. Sci Rep.

[22] Wang S, Zhao H, Li F, Xu Y, Bao H, Zhao D. Higher Chronic Endometritis Incidences within Infertile Polycystic Ovary Syndrome Clinical Cases. J Healthc Eng. 2022, 11;2022:9748041. 10.1155/2022/9748041 PMC9017445 35449841

[23] Jiang X, Cai J, Liu L, Liu Z, Wang W, Chen J (2022). Does conventional morphological evaluation still play a role in predicting blastocyst formation?. Reprod Biol Endocrinol.

[24] Papanikolaou EG, Kolibianakis EM, Tournaye H, Venetis CA, Fatemi H, Tarlatzis B (2008). Live birth rates after transfer of equal number of blastocysts or cleavage-stage embryos in IVF. A systematic review and meta-analysis. Hum Reprod.

[25] Chang HJ, Lee JR, Jee BC, Suh CS, Kim SH (2009). Impact of blastocyst transfer on offspring sex ratio and the monozygotic twinning rate: a systematic review and meta-analysis. Fertil Steril.

[26] Braude P, Bolton V, Moore S (1998). Human gene expression first occurs between the four- and eight-cell stages of preimplantation devel-opment. Nature.

[27] Kaartinen N, Kananen K, Tomás C, Tinkanen H (2017). Day 4 embryos should not be underestimated in IVF. Gynecol Reproduct Endocrinol-UK.

[28] Tao J, Tamis R, Fink K, Williams B, Nelson-White T, Craig R (2002). The neglected morula/compact stage embryo transfer. Hum Reprod.

[29] Holschbach V, Weigert J, Dietrich JE, Roesner S, Montag M, Strowitzki T (2017). Pregnancy rates of day 4 and day 5 embryos after culture in an integrated time-lapse incubator. Reprod Biol Endocrinol.

[30] Fabozzi G, Alteri A, Rega E, Starita MF, Piscitelli C, Giannini P (2016). Morphological assessment on day 4 and its prognostic power in selecting viable embryos for transfer. Zygote.

[31] Li HX, Xu XJ, Liu L (2021). A New Day 4 Grading System to assess embryo quality in frozen embryo transfer cycles. Reprod Sci.

[32] Wang C, Gu Y, Zhou J, Zang J, Ling X, Li H (2022). Leukocyte telomere length in children born following blastocyst-stage embryo transfer. Nat Med.

[33] Hajshafiha M, Oshnouei S, Mostafavi M, Dindarian S, Kiarang N, Mohammadi S (2021). Evaluation of the relationship between serum estradiol levels on human chorionic gonadotropin administration day and intracytoplasmic sperm injection outcomes: a retrospective population-based study. Int J Reprod Biomed.

[34] Bourgain C, Devroey P (2003). The endometrium in stimulated cycles for IVF. Hum Reprod Update.

[35] Zapantis G, Szmyga MJ, Rybak EA, Meier UT (2013). Premature formation of nucleolar channel systems indicates advanced endometrial maturation following controlled ovarian hyperstimulation. Hum Reprod (Oxford England).

[36] Meng F, Zapantis G, Williams SZ, Lieman HJ, Buyuk E, Meier UT (2018). Status of nucleolar channel systems in uterine secretions accurately reflects their prevalence-a marker for the window of implantation-in simultaneously obtained endometrial biopsies. Fertil Steril.

[37] Fayazi M, Beigi Boroujeni M, Salehnia M, Khansarinejad B (2014). Ovarian stimulation by exogenous gonadotropin decreases the implantation rate and expression of mouse blastocysts integrins. Iran Biomed J.

[38] Li R, Wu J, He J, Wang Y, Liu X, Chen X (2017). Mice endometrium receptivity in early pregnancy is impaired by maternal hyperinsulinemia. Mol Med Rep.

[39] Bai X, Zheng L, Li D, Xu Y (2021). Research progress of endometrial receptivity in patients with polycystic ovary syndrome: a systematic review. Reprod Biol Endocrinol.

[40] Sutaji Z, Elias MH, Ahmad MF, Karim AKA, Abu MA (2022). A Systematic Review and Integrated Bioinformatic Analysis of Candidate Genes and pathways in the endometrium of patients with polycystic ovary syndrome during the Implantation window. Front Endocrinol.

[41] Zhang J, Ding N, Xin W, Yang X, Wang F (2022). Quantitative proteomics reveals that a Prognostic signature of the Endometrium of the polycystic ovary syndrome women based on ferroptosis proteins. Front Endocrinol.

[42] Wood JR, Dumesic DA, Abbott DH, Strauss JF (2007). Molecular abnormalities in oocytes from women with polycystic ovary syndrome revealed by microarray analysis. J Clin Endocrinol Metab.

[43] Sundvall L, Kirkegaard K, Ingerslev HJ, Knudsen UB (2015). Unaltered timing of embryo development in women with polycystic ovarian syndrome (PCOS): a time-lapse study. J Assist Reprod Genet.

[44] Chappell NR, Barsky M, Shah J, Peavey M, Yang L, Sangi-Haghpeykar H (2020). Embryos from polycystic ovary syndrome patients with hyperandrogenemia reach morula stage faster than controls. Fertil Steril Rep.

[45] Ying Y, Lu X, Zhang H, Arhin SK, Hou X, Wang Z (2021). Clinical and perinatal outcomes of fresh single-blastocyst-transfer cycles under an early follicular phase prolonged protocol according to day of trigger estradiol levels. PeerJ.

[46] Zhang W, Tian Y, Xie D, Miao Y, Liu J, Wang X (2019). The impact of peak estradiol during controlled ovarian stimulation on the cumulative live birth rate of IVF/ICSI in non-PCOS patients. J Assist Reprod Genet.

